# A Rare Case of Blood Culture-Negative Isolated Pulmonic Valve Endocarditis Causing Empyema

**DOI:** 10.7759/cureus.29137

**Published:** 2022-09-13

**Authors:** Bradley Casey, Destinee A Hua, Amudhan Jyothidasan, Manoj Bhandari

**Affiliations:** 1 Internal Medicine, Cape Fear Valley Medical Center, Fayetteville, USA; 2 Cardiology, Cape Fear Valley Medical Center, Fayetteville, USA

**Keywords:** blood culture-negative endocarditis, echocardiography, pericardial effusion, poor oral hygiene, empyema, pulmonic valve endocarditis

## Abstract

Right-sided native valve infective endocarditis (IE) refers to IE involving the tricuspid or pulmonic valve. The most common factors causing right-sided IE include intravenous drug use, intracardiac device, and central venous catheters. Isolated pulmonic valve IE has only been reported in less than 2% of all IE cases. We present a unique case of a patient with a history of poor oral hygiene found to have isolated blood culture-negative pulmonic valve IE, who subsequently developed empyema positive for *Streptococcus constellatus*.

## Introduction

Infective endocarditis (IE) has several pathogeneses including endothelial damage, platelet adhesion, and microbial adherence to valvular tissue. Complications of endocarditis include but are not limited to congestive heart failure, embolization, renal dysfunction, and abscess formation [1]. Here, we present a rare case involving a male with poor dental hygiene presenting to the emergency department with the chief complaint of generalized weakness and fatigue. Incidentally, he was found to have an empyema and concerns for a large pulmonary embolism coming from the pulmonic valve. Transthoracic echocardiography (TTE) was obtained, which demonstrated large vegetation as opposed to a pulmonary embolism. Further workup determined that the patient developed empyema from pulmonic valve endocarditis likely due to his poor oral hygiene.

## Case presentation

**Figure 1:**
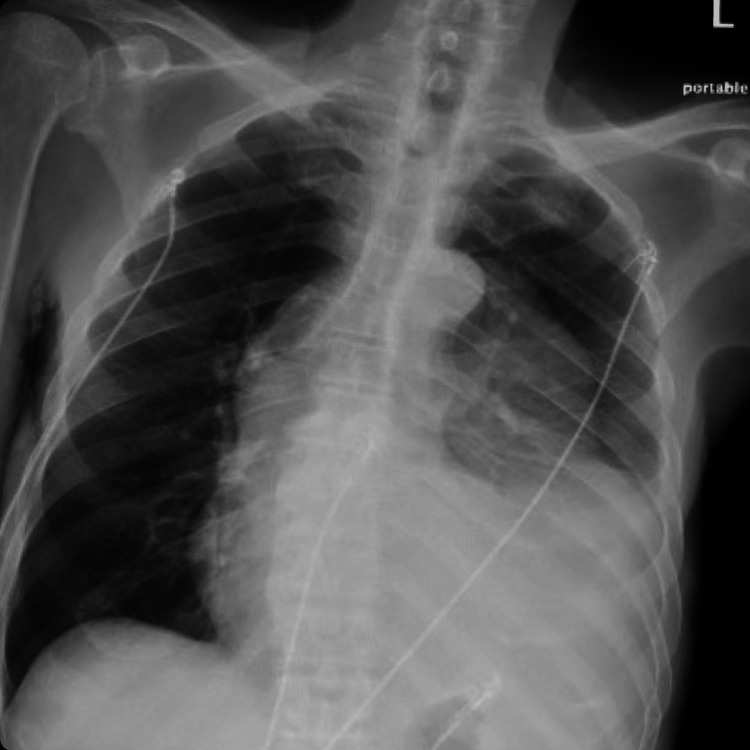
Chest X-ray showing pneumonia as well as abnormal mass in the left suprahilar region

A 73-year-old male with a past medical history of dementia, hypertension, and hyperlipidemia presented to the emergency department (ED) by family due to increasing generalized weakness, fatigue, and poor oral intake over the past three months. Over the last month, the patient experienced persistent cough, shortness of breath, and subjective fevers. Family members were concerned due to more than 40 pounds of unintentional weight loss over a three-month period. They also reported that over the last four months, the patient had lost approximately four teeth due to poor oral hygiene. When the patient initially arrived at the ED, he was tachycardic in the low 100s, hypothermic at 92.6°F/33.7°C, tachypneic in the low 20s, and hypotensive at 96/52. Chest X-ray was concerning for pneumonia as well as an abnormal mass in the left suprahilar distribution (Figure 1).

**Figure 2:**
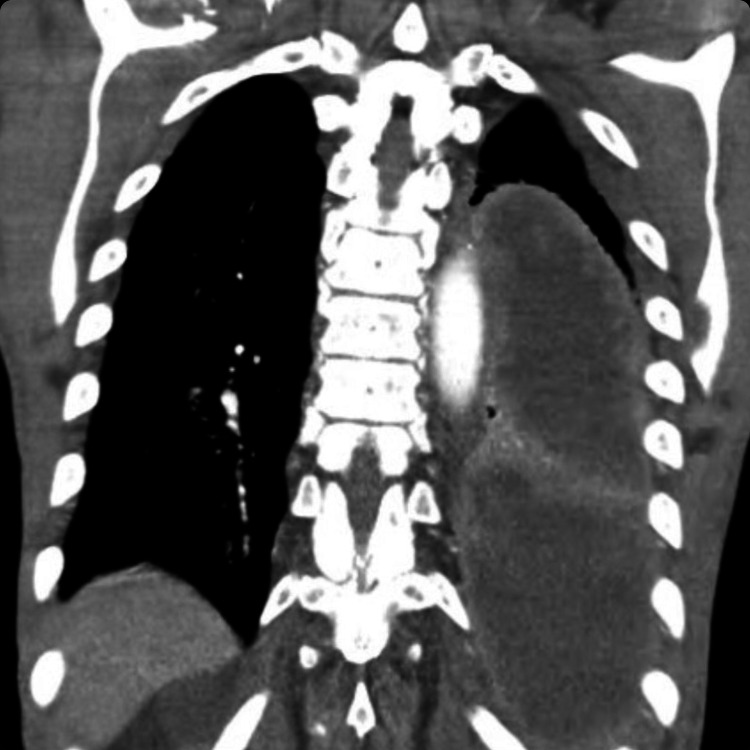
CT scan of the chest showing large left thoracic fluid collection (20 x 13 x 10 cm)

**Figure 3:**
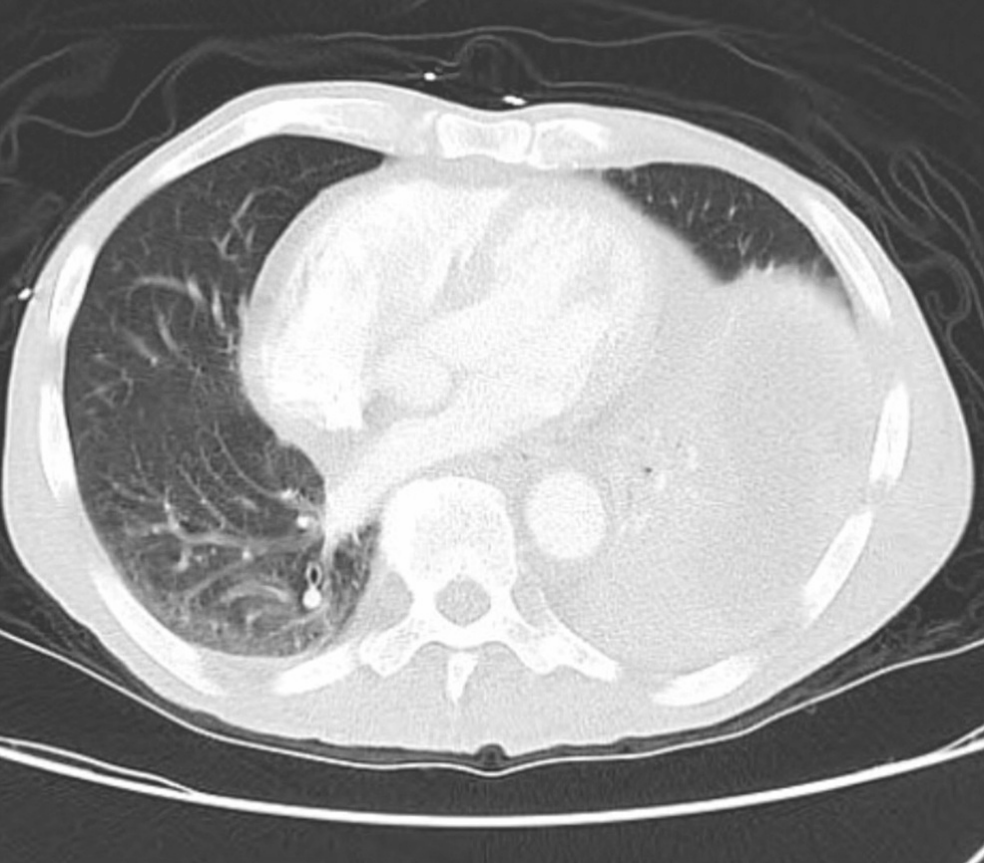
CT scan showing large pleural effusion and large abnormality, which was concerning for a pulmonary embolism arising from the pulmonic valve

**Figure 4:**
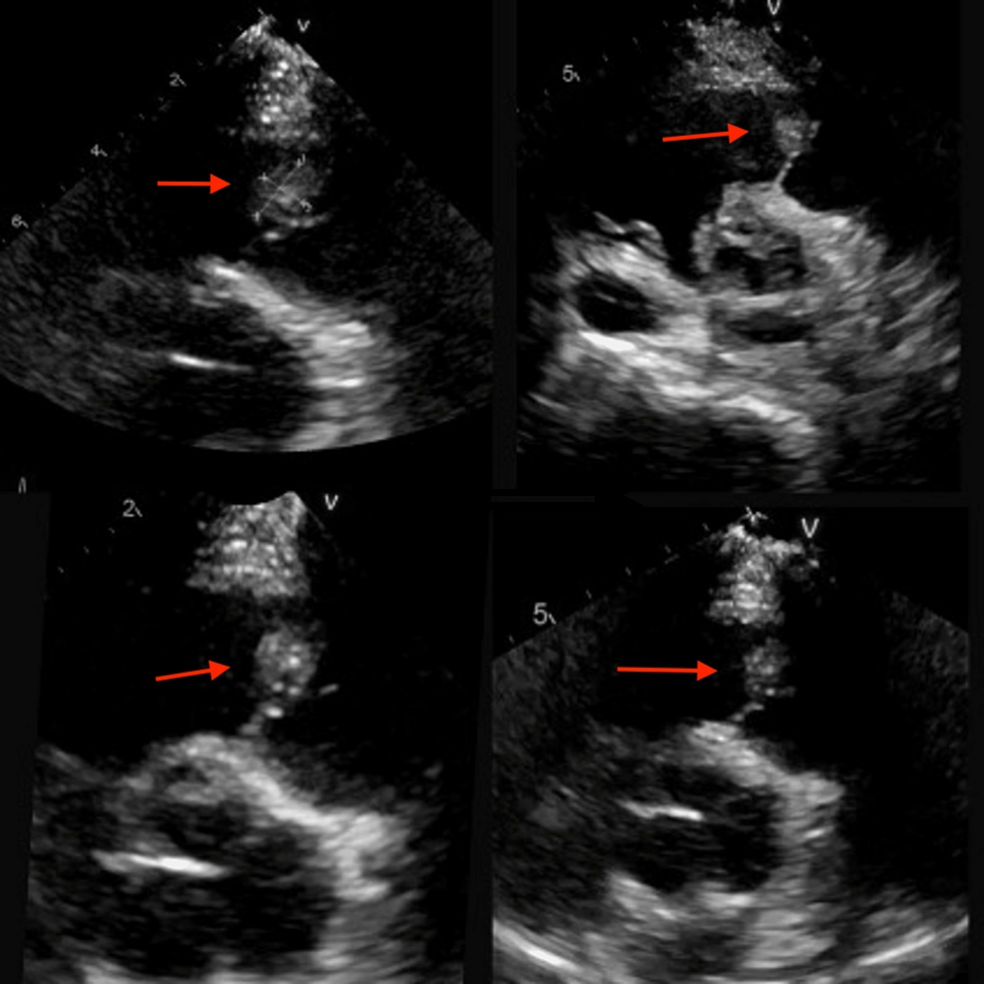
Multiple views from transthoracic echocardiography showing pulmonic valve vegetation measuring 1.29 x 0.89 cm

CT chest with contrast showed a large left thoracic fluid collection (20 x 13 x 10 cm) as well as a large abnormality, which was concerning for a pulmonary embolism arising from the pulmonic valve (Figures 2, 3). A heparin drip was initiated due to concern for pulmonary embolism. Blood cultures from the ED showed no growth. Inflammatory markers were elevated including sedimentation rate greater than 130 mm/hour (normal: 0-20 mm/hour) and C-reactive protein of 159 mg/L (normal: less than 3.0 mg/L). Workup included TTE, which showed pulmonary valve vegetation measuring 1.29 x 0.89 cm, a small pericardial effusion, and mild pulmonic valve regurgitation without any involvement of other heart valves (Figure 4). Cardiology was consulted and recommended discontinuing heparin as this was pulmonic valve endocarditis. Interventional radiology was consulted for an ultrasound-guided thoracentesis with the removal of 40 mL of purulent fluid and placement of a pleural drain. The pleural fluid culture was positive for *Streptococcus constellatus*. Blood cultures remained negative during the hospital course. The patient developed persistent fever for a week, which was attributed to septic microembolization of the lung. The patient's antibiotics from the ED of Rocephin and azithromycin were changed to cefepime and vancomycin. Infectious disease as well as pulmonology concluded that the empyema resulted from the pulmonic valve endocarditis. Cardiothoracic surgery was consulted and believed the patient was too high risk for intervention; therefore, antibiotics were continued on an outpatient basis. The patient was discharged to a rehab facility on six weeks of intravenous ceftriaxone.

## Discussion

The overall incidence of IE is 1-10 per 100,000 patients per year, mainly affecting older individuals. Right-sided endocarditis is less common than left-sided, and only affects approximately 5-10% of all IE cases. The tricuspid valve is the most common right-sided valve that is involved. Isolated pulmonary valvular endocarditis (PVE) has only been reported to occur in less than 2% of all IE [2]. Blood culture-negative IE (BCNIE) occurs when no causative microorganism can be grown using routine blood culture methods. BCNIE accounts for 5-10% of all cases of endocarditis. A European study that included 820 cases indicated that 20% of patients with confirmed IE had negative blood cultures [3].

PVE rarity has been attributed to multiple factors including lower pressure within the right heart, lower incidence of congenital malformations or acquired valvular abnormalities, lower oxygen content of venous blood, and the differences in the endothelial lining and vascularization of the valve [4]. The most common risk factor for PV endocarditis is intravenous (IV) drug abuse. Other risk factors have also been reported including male gender, central venous catheters, alcoholism, dental extraction, gonorrhea, liver or renal transplantation, bowel surgery, colonic angiodysplasia, and congenital heart disease (CHD) [4,5]. Around 28% of PVE cases have been reported with no identifiable predisposing factors [6].

Right-sided IE most commonly presents with persistent fever, cough, and dyspnea, which is secondary to septic emboli [6]. TTE has been shown to have a diagnostic yield of around 91% to diagnose PVE [4]. Transesophageal echocardiogram (TEE) may not have a diagnostic yield as high as TTE due to the pulmonic valve being on the anterior aspect and farthest from the TEE probe [7]. Positron emission tomography-computed tomography (PET-CT) imaging has been proposed to have some significance in diagnosing and determining the extent of PVE [3].

Our patient lost several teeth over the previous months leading to admission due to the severity of his dental hygiene. Pleural fluid cultures with the growth of an organism known to be part of our normal oral flora with the degree of the patient’s dental disease led us to conclude poor dentition was likely the cause of his endocarditis. The mitral valve is the most common valve affected by dental origin endocarditis [8]. In this case, we witnessed pulmonic valve endocarditis from our patient with dental disease. Furthermore, empyema caused by BCNIE pulmonic valve endocarditis has never been reported to our knowledge. Many studies have reported empyema and lung abscess caused by tricuspid valve endocarditis, but cultures were not reported in those studies [9].

## Conclusions

We present a case, to our knowledge, which has never been reported before. This is a case of BCNIE of the pulmonic valve that led to an empyema. The culture from the pleural fluid grew *Streptococcus constellatus*, an organism that is part of our normal oral flora. Multiple consulting specialists agreed that the patient’s endocarditis likely originated from poor dental hygiene. When investigating differential diagnoses for causes of empyema, a TTE should be considered as part of the workup. Pleural fluid should always be cultured, especially in the setting of negative blood cultures to help guide antimicrobial therapy. From our case, we propose additional diagnostic testing to be considered in patients with empyema of unknown origin with risk factors or high clinical suspicion for endocarditis.
